# Status of gastrointestinal nematode infections and associated epidemiological factors in sheep from Córdoba, Colombia

**DOI:** 10.1007/s11250-022-03170-2

**Published:** 2022-04-26

**Authors:** Elisa Brunal Tachack, Teresa Oviedo-Socarrás, Misael Oviedo Pastrana, Luis Carlos Pérez-Cogollo, Yonairo Herrera Benavides, Clara Rugeles Pinto, Oscar Vergara Garay

**Affiliations:** 1 Departamento de Ciencias Pecuarias, Facultad de Medicina Veterinaria y Zootecnia, Universidad de Córdoba, Montería, Colombia; 2 Corporación Colombiana de Investigación Agropecuaria - Agrosavia, Centro de Investigación Turipaná, Cereté, Córdoba, Colombia

**Keywords:** Helminthiasis, Epidemiology, Parasite burden, Ovine, Colombia

## Abstract

Gastrointestinal nematodes cause serious economic losses in sheep production systems. To determine the prevalence and risk factors associated with these parasites, a study was conducted on 595 sheep in Córdoba, Colombia. Prevalence and parasite burden were determined using the McMaster technique. Larvae were cultured from feces to identify the nematode genera. For the analysis of associated factors and parasite burden, data means, medians, and confidence intervals were compared. A georeferenced data analysis was performed and an epidemiological map was constructed. An overall prevalence of 88.2% was found, with the highest prevalence and parasite burden for Strongylida (83.2%) and *Strongyloides* (41%) nematodes. The mean parasite burden was 1255 eggs per gram. The gastrointestinal nematode genera identified were *Trichostrongylus*, *Strongyloides*, *Haemonchus*, *Oesophagostomum*, *Bunostomum*, and *Cooperia*. In general, a significant association was found between parasite burden and dewormed animals, anthelmintic used, date of last deworming, and breeds. The FAMACHA® method and body condition showed a significant association with the Strongylida parasite burden. Clusters with higher intensity of gastrointestinal nematode burden and high prevalence were observed in the regions of Bajo Sinú, Sinú Medio, and San Jorge.

## Introduction

Sheep (*Ovis aries*) are a susceptible animal species to multiple gastrointestinal nematodes (GIN). Infections can be associated with several factors such as animal’s age, breed, parasite species involved, and degree of parasitic infection (Williams et al. 2021). Nematode infections can alter animal walefare, therefore, they can reduce the productive leves of farms, regarless of the production system (Herrera et al. 2013).

GIN causes a major health and economic impact on extensive livestock production due to its negative effect on performance parameters like weight gain and mortality, as well as the high costs of anthelmintic treatments. Additionally, the emergence of GIN populations resistant to all anthelmintic families available in the Americas makes the control of these parasites even more difficult (Kaplan 2020).

Parasitic gastroenteritis is a multi-etiological disease; however, the GIN responsible for sheep mortality belongs mainly to the order Strongylida, superfamily Strongyloidea (Zajac and Garza 2020). Among these nematodes, the genera *Haemonchus*, *Trichostrongylus*, *Teladorsagia* (*Ostertagia*), and *Oesophagostomum* are the most frequent (Torres-Acosta et al. 2012; Herrera et al. 2013). Other GIN belonging to different taxonomic orders that commonly parasitize sheep in the Americas include *Strongyloides*, *Aoncotheca* (formerly *Capillaria*), *Trichuris*, and *Skrjabinema*, although these nematodes are not considered to be of major pathogenic importance and cause disease only in unusual circumstances (Zajac and Garza 2020).

In Colombia, studies on parasitic gastroenteritis have revealed the prevalence of endoparasites in different regions and climates involved in sheep farming (Herrera et al. 2013; Pinilla et al. 2019). However, little is known about the prevalence of GIN infections and their associated factors in sheep farms across different regions of the department of Córdoba.

For the rational and sustainable control of gastrointestinal parasitism in sheep, a thorough knowledge of the parasites’ epidemiology and their interaction with the host in a specific environment is required (Keyyu et al. 2005). Therefore, knowledge of the parasite species found in a specific region, their prevalence, degree of infection, characteristics of the local climate, average flock size, and local management practices are considered essential information (Kaplan and Vidyashankar 2012). For this reason, the objective of this study was to determine the prevalence and risk factors associated with gastrointestinal parasitism in sheep from the department of Córdoba in Colombia.

## Materials and methods

### Study location

The study was carried out in the department of Córdoba, Colombia. This department is located in a lowland tropical ecosystem, which is divided into six regions, as follows: Alto Sinú, Sinú Medio, Bajo Sinú, Sabanas, San Jorge, and Costanera. On average, it has an altitude of 30 m.a.s.l., an annual temperature of 28°C, a relative humidity of 82%, and 1,400 mm of precipitation. It belongs to the tropical rainforest climate formation and during the year there is a rainy season (May to November) and a dry season (December to April); the rainfall increases from north to south.

### Study design

A descriptive cross-sectional study was carried out to determine the prevalence and associated factors of GIN in sheep from the department of Córdoba. To calculate the sample size of the study, the following assumptions were considered: an infinite population of sheep, 75% estimated true prevalence, 80% sensitivity, 90% diagnostic specificity, a precision of 6%, and a confidence level of 95%. The calculation determined a sample size of 511 sheep. In the end, 595 sheep were sampled in 60 farms.

On each farm, one breeding male, three breeding females, two rearing females, two rearing males, and one lamb from each sex were sampled. The animals sampled in each farm were randomly selected. The study was conducted over a 12-month period.

### Sample and data collection

Fecal samples were taken directly from the rectum using polyethylene gloves and blood samples were taken from the jugular vein using vacutainer tubes with anticoagulant. The samples were identified and kept refrigerated at 4°C until processing.

In addition, a clinical examination was performed on each animal, recording the aspects of the breed, sex, age, productive stage, weight, assessment of ocular conjunctival coloration by the FAMACHA® method, and assessment of body condition. An epidemiological survey on aspects related to the management and control of gastrointestinal parasites in the flock was conducted with each producer. The geographic coordinates of each farm were determined by Global Positioning System (GPS).

### Laboratory testing

Individual counts of egg per gram of feces (EPG) were determined using the McMaster technique with a sensitivity of 50 EPG. To identify the GIN genera, a fecal pool copro-culture from each flock was carried out following the procedure described by Taylor et al. (2016). Larvae were identified by microscopy using taxonomic keys based on their morphology: total size, tail size, the shape of the sheath, size and shape of the esophagus, and number and shape of intestinal cells and refractile bodies (Van Wyk et al. 2004).

### Data analyses

The EPG per animal, the prevalence of GIN infection per flock, and total sheep population were determined. Deworming, anthelmintic used, time elapsed since the last deworming, sex, productive stage, and breed were variables analyzed to see their relation with the parasitic burden. The breeds were grouped as Colombian hair sheep (OPC), including Criollo, Chino Rojo, Sudan, and Abisinio; and breeds not belonging to Colombian hair sheep (Non-OPC), among them Katahdin, Santa Inés, Pelibuey, Black Belly, Dorper, Persian, White Dorper, and some crossbreeds with OPC. The association of the different variables was made with the overall parasitic burden and with the burden of each type of nematode. Also, clinical factors were associated with GIN burden in sheep.

For the analysis of risk factors associated with the parasitic burden, data means, medians, and confidence intervals were compared. The Kruskal-Wallis test was applied with a significance level of 5%, and the statistical program EpiInfo version 7.2.2.2.2 was used. Finally, areas with the highest concentration of parasite burdens were identified using the kernel density estimator. The georeferenced data were analyzed and an epidemiological map was constructed using the standard deviation of the mean in the categorization of the data and a bandwidth of 25 km. The QGIS program, version 3.4, was used for this purpose.

## Results

### Prevalence and parasite burden

An 88.2% of the animals were positive for one or more groups of parasites. An 83.2% were positive for nematodes of the order Strongylida, 41.0% for *Strongyloides* spp., and 2.2% for *Trichuris* spp. (Table 1). On the farm scale, the prevalence was 100%. Overall parasite burden showed an average count of 1255 EPG; the Strongylida order showed the highest average parasite burden (943 EPG), followed by *Strongyloides* spp. and *Trichuris* spp*.* with low average parasite burdens (Table 1). Multiple parasitic infections were frequently observed in the animals under study. Co-infections of Strongylida and *Strongyloides* spp. were more frequently found among sheep. The composition of GIN infections is presented in Table 2.

**Table 1:** Prevalence of gastrointestinal nematode infections and parasite burdens in sheep in the department of Córdoba

Variable	*n*	Prev. (%)	EPG mean	Inf.	Sup.	Sd
Strongylida	595	83.2	943	806	1079	1698
*Strongyloides* spp.	595	41.0	311	227	395	1043
*Trichuris* spp.	595	2.2	2	1	3	13
Overall count	595	88.2	1255	1093	1418	2021

*n*, sampled animals; *Prev.*, prevalence; *EPG*, eggs per gram; *Inf.*, lower limit; *Sup.*, upper limit; *Sd*, standard deviation

**Table 2:** Gastrointestinal nematode infection and co-infection in sheep in the department of Córdoba

Parasite	Multiple infections				Negatives
	I	II		III	
		*Strongyloides* spp.	*Trichuris* spp.	*Strongyloides* spp. *Trichuris* spp.	
Strongylida	276	206	5	8	
*Strongyloides* spp.	30	-	-	-	70
*Trichuris* spp.	0	-	-	-	
Total	306	211		8	70
	(51.4%)	(35.5%)		(1.3%)	(11.8%)

*I*, animals infected with one type of nematode; *II*, animals co-infected with two types of nematodes; *III*, animals co-infected with three types of nematodes

### Predisposing factors associated with GIN burden in sheep

Overall, an association was found between the parasitic burden and the variables: dewormed animal, anthelmintic used, last deworming, and breed (Table 3). In terms of nematode types, the parasite burden of the order Strongylida was significantly associated with the variables: dewormed animal, anthelmintic used, time elapsed since the last deworming, and breed. For *Strongyloides* spp., an association was identified with the variables: dewormed animal, time since the last deworming, and productive stage. In the case of *Trichuris* spp., an association was only found with the productive stage of the animal (Table 4).

**Table 3:** Factors associated with overall gastrointestinal nematode burden in sheep in the department of Córdoba, Colombia

Variable	Category	Sample size	Pos	Mean (EPG)	Sd.	Med.	*p*-value
Dewormed animal	Yes	298	269	1583	2459	700	
	No	297	257	926	1382	450	
Anthelmintic used	Fenbendazole	207	190	1677	2525	900	0.0485
	Ivermectin	28	27	2530	3595	800	
	Levamisole	54	43	881	1158	500	
	Natural med.	8	8	669	476	550	
Last deworming	<45 days	138	122	1879	2839	800	0.0007
	46–120 days	160	147	1328	2052	600	
	>120	297	257	926	1382	450	
Sex	Female	368	325	1170	1860	525	0.204
	Male	227	201	1392	2255	650	
Productive stage	Lambs	134	112	1349	2576	500	0.0750
	Rearing lambs	201	183	1403	2114	700	
	Pregnant ewes	64	59	670	728	400	
	Lactating ewes	98	87	1472	1925	725	
	Empty ewes	33	29	932	1233	400	
	Breeding male	65	56	1019	1620	500	
Breed	OPC	292	261	949	1210	525	0.0478
	Non OPC	303	265	1550	2538	650	

*Pos*, positives; *EPG*, eggs per gram; *Sd*, standard deviation; *Natural Med.*, use of medicinal plants; *OPC*, Colombian hair sheep; *Med.*, median

**Table 4:** Factors associated with Strongylida, *Strongyloides* spp., and *Trichuris* spp. burdens in sheep in the department of Córdoba

Variable	Category	*n*	Strongylida				*Strongyloides* spp.				*Trichuris* spp.			
			Pos.	Mean (EPG)	Med. (EPG)	*p*-value	Pos.	Mean (EPG)	Med. (EPG)	*p*-value	Pos.	Mean (EPG)	Med. (EPG)	*p*-value
Dewormed animal	Yes	298	259	1205	500	0.000	103	377	0	0.018	6	2	0	0.773
	No	297	236	680	300		140	245	0		7	2	0	
Anthelmintic used	Fenbendazole	207	184	1385	600	0.016	74	290	0	0.251	6	2	0	0.448
	Ivermectin	28	26	1238	375		9	1293	0		0	0	0	
	Levamisole	54	40	594	350		19	286	0		0	0	0	
	Natural med.	8	8	669	550		0	0	0		0	0	0	
Last deworming	< 45 days	138	118	1537	550	0.000	38	342	0	0.007	0	0	0	0.085
	46–120 days	160	141	918	500		65	407	0		6	3	0	
	> 120 days	297	236	680	300		140	245	0		7	2	0	
Sex	Female	368	308	932	350	0.676	143	237	0	0.053	8	1	0	0.968
	Male	227	187	961	400		100	430	0		5	2	0	
Productive stage	Lambs	134	102	1074	275	0.306	56	274	0	0.000	0	0	0	0.029
	Rearing lambs	201	174	911	450		101	488	50		9	4	0	
	Pregnant ewes	64	55	604	350		16	65	0		1	1	0	
	Lactating ewes	98	82	1165	400		39	307	0		1	1	0	
	Empty ewes	33	28	867	400		8	62	0		2	3	0	
	Breeding male	65	54	805	350		23	215	0		0	0	0	
Breed	OPC	303	243	729	300	0.041	136	216	0	0.070	9	3	0	0.137
	Non OPC	292	252	1147	450		108	401	0		4	1	0	

*n*, number of observations; *Pos.*, positives; *Med.*, median; *EPG*, eggs per gram

### Clinical factors associated with GIN burden in sheep

The parasite burden of the sheep studied was related to clinical parameters, including body condition, weight, heart rate, respiratory rate, temperature, hematocrit, and FAMACHA® score. Strongylida nematode burden was associated with sheep body condition, hematocrit, and FAMACHA® score, while *Strongyloides* spp*.* parasite burden was associated with body condition and hematocrit. *Trichuris* spp*.* parasite burdens were not associated with any of the variables studied (Table 5).

**Table 5:** Clinical factors associated with Strongylida, *Strongyloides* spp., and *Trichuris* spp. burdens in sheep in the department of Córdoba

Variable	Category	*n*	Strongylida				*Strongyloides spp.*				*Trichuris spp.*			
			Pos.	Mean (EPG)	Med. (EPG)	*p*-value	Pos.	Mean (EPG)	Med. (EPG)	*p*-value	Pos.	Mean (EPG)	Med. (EPG)	*p*-value
Body condition	Good	178	141	694	300	0.018	66	220	0	0.022	3	1	0	0.324
	Average	296	252	984	500		136	402	0		9	2	0	
	Bad	120	101	1208	400		41	224	0		1	0	0	
Hcrit.	High	149	130	650	350	0.001	57	201	0	0.028	0	0	0	0.056
	Average	294	231	808	300		113	327	0		7	2	0	
	Low	147	129	1514	500		71	391	0		6	3	0	
Famacha®	1	38	30	499	200	0.000	15	116	0	0.526	1	4	0	0.555
	2	242	197	607	300		95	290	0		5	2	0	
	3	216	181	841	400		85	290	0		3	1	0	
	4	90	78	1685	600		45	443	25		4	3	0	
	5	9	9	6867	5500		3	872	0		0	0	0	

*n*, number of observations; *Pos.*, positives; *EPG*, eggs per gram; *Med.*, median; *Hcrit.*, hematocrit

### Geographical factors associated with GIN burden in sheep

**Fig. 1:**
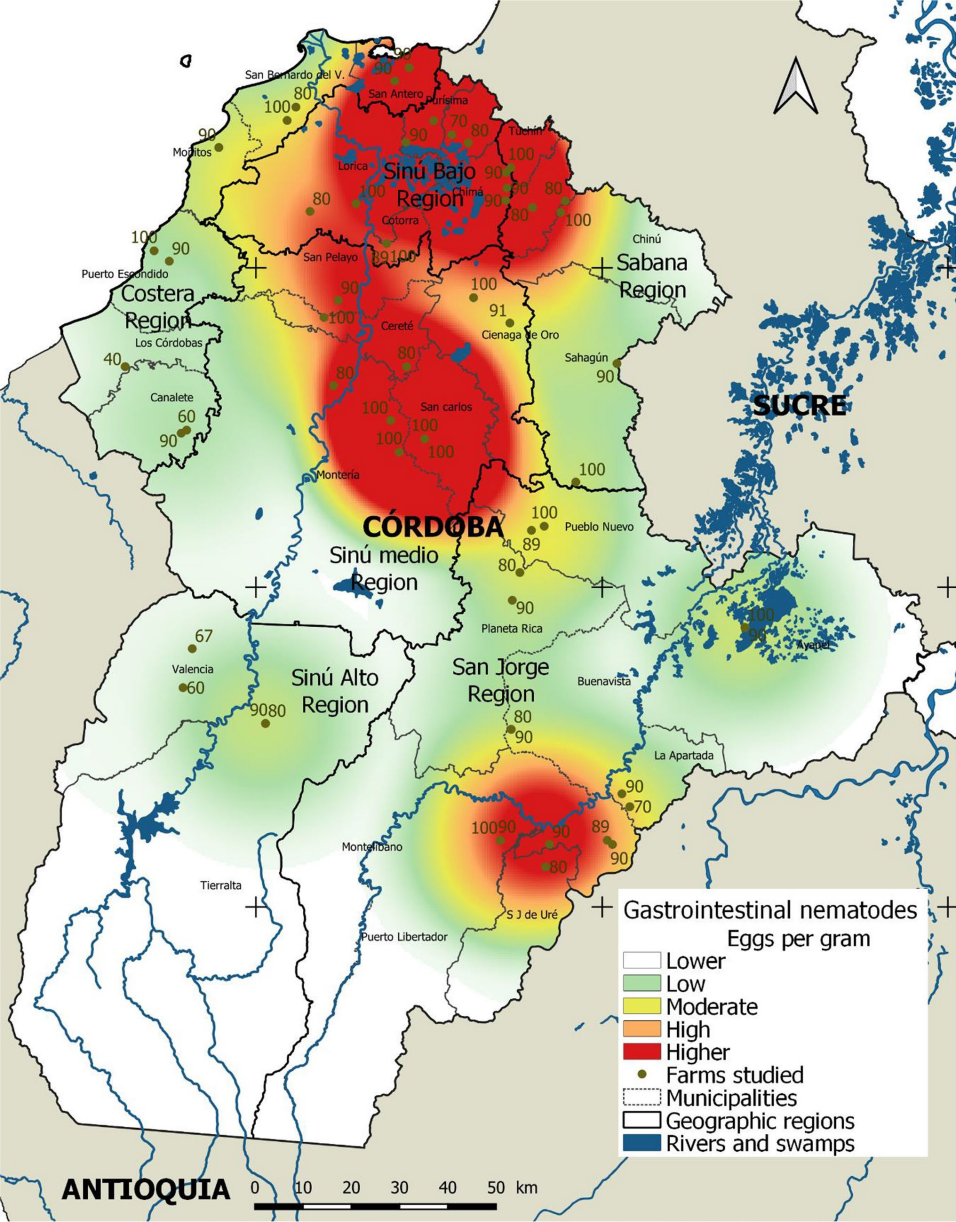
Epidemiological map of gastrointestinal nematode infection prevalence and burden in sheep in the department of Córdoba. Categorization is shown using standard deviation over the mean of the data represented

An epidemiological map was generated, which depicts the association between parasite burdens and their distribution between regions and municipalities in the department of Córdoba (Fig. 1). The map shows the location of the farms evaluated with their respective GIN prevalence. Two high-density clusters can be observed; the first one is located between Sinú Medio and Sinú Bajo regions and extends towards the north of the Sabana region, and the second one in the San Jorge region. The municipalities with the highest parasite burdens were Purísima, Lorica, Cereté, San Carlos, Puerto Libertador, and San José de Uré.

### Genera of nematodes identified

In the farms studied, a 100% prevalence was identified for *Trichostrongylus* spp., 78.3% for *Strongyloides* spp., 76.7% for *Haemonchus* spp., 53.3% for *Oesophagostomum* spp., 10% for *Bunostomun* spp., and 1.7% for *Cooperia* spp. The results of the fecal culture and the percentage of infective larvae in each genus are presented in Table 6.

**Table 6:** Infective larvae prevalence and percentages of the nematode genera identified in sheep farms in the department of Córdoba

Genus	Farms	Prevalence per farm (%)	Infective larvae percentage (L3) ± SD
*Trichostrongylus* spp.	60	100	52.0±21
*Strongyloides* spp.	47	78.3	18.6±16.5
*Haemonchus* spp.	46	76.7	17.4±16
*Oesophagostom* spp.	32	53.3	11.4±16
*Bunostomun* spp.	6	10.0	0.5±1.7
*Cooperia* spp.	1	1.7	0.1±0.5

*Obs.*, number of observations; *Pos.*, positives; *Sd.*, standard deviation; *Med.*, median

## Discussion

This study reveals a serious situation of GIN parasitism in sheep farms in the Colombian lowland tropics. Although the parasitic infection was expected due to an apparent predisposition of sheep in grazing systems, it is important to highlight that the seriousness of this finding lies not only in the high prevalence of parasitism but also in the high parasite burden, which averaged 1255 EPG/animal. This value is considered a high-level infection, taking into account the classification of infection levels according to Hansen and Perry (1994). Considering the parasitic groups, high-level infections were found to be caused by Strongylida nematodes, mostly from the Trichostrongylidae family, which harbors genera of pathogenic importance in sheep such as *Haemonchus contortus* (Wang et al. 2020) and whose parasitic burdens in this study showed a significant association with clinical factors such as body condition, hematocrit, and FAMACHA score. *Strongyloides* spp. infections were considered moderate and *Trichuris* spp. infections low.

Dewormed animals and those being recently dewormed before the McMaster test presented the highest Strongylida and *Strongyloides* spp*.* burdens. This contradictory situation may be explained as a desperate need of producers to apply anthelmintic treatments due to the inefficient reduction of parasite burdens. In the study, we observed that different farms performed monthly applications of anthelmintics; these results suggest signs of anthelmintic resistance, especially related to the infections due to Strongylida nematodes.

Regardless of the type of anthelmintic used, parasite burdens were high, suggesting a possible inefficiency of fenbendazole, ivermectin, levamisole, and the use of natural medicine in the control of parasitism, although it should be noted that lower Strongylida and overall egg counts were found in sheep that had been treated with levamisole. The development of anthelmintic resistance, especially to ivermectin and fenbendazole, has been previously reported in several studies (Torres-Acosta et al. 2012). This result reflects the need to implement monitoring programs to assess the efficacy of anthelmintics used in the parasitic control of sheep.

The degree of infection by *Trichuris* spp. did not differ according to the type of anthelmintic used. It is necessary to point out that burdens of this parasitic group were very low, which cannot suggest the effectiveness of the anthelmintics used since there was no significant difference found. This result is rather associated with the low prevalence and low burdens of *Trichuris* spp. in the department of Córdoba.

The GIN infection prevalence and parasite burdens were similar in males and females. Most studies address the influence of sex on GIN prevalence and in general, there is no consensus on which sex is more affected (Poddar et al. 2017).

Productive stage was not associated with overall parasite burdens or Strongylida infections. However, there is a trend of higher burdens in rearing animals and lactating ewes. In that sense, several studies agree that young animals are more susceptible to gastrointestinal parasitism (Raza et al. 2007) since they have an immune system with insufficient development to modulate parasitic infestations (Khan et al. 2010). On the other hand, it is known that regardless of the year, season, and age, ewes present greater susceptibility to GIN during lactation (González-Garduño et al. 2014). Experimentally, there are studies that support the hypothesis that full-grown animals can acquire immunity against GIN (Knox 2000).

The breed was associated with overall GIN parasite burden and particularly for Strongylida and *Strongyloides* spp. nematodes. It was observed that breeds belonging to OPC had lower parasite burdens. Breeding studies of small ruminants have revealed a reduction of EPG in feces when animals that are naturally resistant to GIN infection are selected for breeding (Eady et al. 1996). Genetic factors are known to contribute to the ability of sheep to cope with the challenge of intestinal parasitism; some studies have shown that different breeds of sheep exhibit varying resistance to intestinal parasite infection (Aboshady et al. 2020). In particular, Preston and Allonby (1979) demonstrated an ascending order of susceptibility in the Red Masai, Blackhead Persian, Merino, Dorper, Corriedale, and Hampshire breeds. However, other undesirable traits, especially in terms of productivity, could make parasitism-resistant breeds unattractive to producers (Woolaston and Baker 1996). In this regard, more recent studies have shown that genome-wide selection strategies can improve the selection of animals with a view on production aspects and helminthiasis resistance traits (McManus et al. 2014).

The clinical variables analyzed, such as body condition, hematocrit, and FAMACHA®, were especially related to high parasite burdens by Strongylida. Animals with the highest degrees of infection showed fair or poor body condition, low hematocrit values, and higher FAMACHA® results. For Strongylida nematodes, the FAMACHA® method had a better predictive value, followed by hematocrit and body condition. *Strongyloides* spp. infections were correlated only with hematocrit and body condition. The above allows us to state that the alteration of the clinical variables and their use as predictive values of the parasitic burden will depend on the parasitic group that is affecting the animals.

The most prevalent nematode genera are associated with the percentage of larvae identified from the fecal cultures. *Trichostrongylus* spp., *Strongyloides* spp., and *Haemonchus* spp. were the most prevalent genera in sheep in the department of Córdoba. These have been previously reported in Colombia (Herrera et al. 2013). It is important to highlight that it was found for the first time in the department of Córdoba that nematodes from the species *Bunostomum* contributed to GIN infections in sheep in this region. Although they were only identified in 10% of the flocks studied, their hematophagous behavior, as well as that of *H. contortus*, causes a decrease in hematological values, including hemoglobin, with immediate consequences such as hypoproteinemia and a decrease in body condition. Analyzing these clinical aspects is important when making decisions related to the deworming management program in sheep production systems.

Besier et al. (2016) indicate that a good body condition may indicate the ability of sheep to better cope with parasitism. Increasing resistance to anthelmintics has resulted in developing alternative control strategies, which reduce the selection of resistant parasites. In this regard, selective deworming programs could be considered within the strategies implemented in the flocks to delay the development of resistance (Van Wyk and Bath 2002). In the present study, it is evident that the FAMACHA® method and the measurement of body condition can be used to establish selective deworming programs in flocks in the department of Córdoba since a significant association with parasite burden was found.

Gastrointestinal parasitism in sheep is a worldwide issue, which requires a deeper understanding of epidemiological aspects such as prevalence, distribution, and seasonal patterns of transmission in different climatic zones. There is a strong belief that climate change may alter the geographical distribution of parasites and their impact on hosts, a situation that is also attributed to the phenomenon of resistance (Charlier et al., 2014). It is believed that climatic change would have profound effects on the epidemiology of parasites, especially for those whose developments outside the definitive host are sensitive to temperature and humidity linked to rainfall. The influence of environmental conditions is particularly evident in spatial distribution studies, allowing the visualization of clusters where animals show not only the prevalence of gastrointestinal parasitism but also high levels of infection. The latter parameter is very interesting to predict the damage caused to individuals and the economic losses of the producer.

The study presents a serious situation of GIN parasitism in sheep farms and suggests a possible resistance to anthelmintics used by the producers, a situation that requires further evaluation. The identification of two high density clusters allows directing epidemiological intervention in the studied region.

## Data Availability

The datasets generated during the current study are available from the corresponding author on reasonable request.

## References

[CR1] Aboshady HM, Stear MJ, Johansson A, Jonas E, Bambou JC (2020). Immunoglobulins as Biomarkers for Gastrointestinal Nematodes Resistance in Small Ruminants: A systematic review. Scientific Reports.

[CR2] Besier RB, Kahn LP, Sargison ND, Van Wyk JA (2016). Diagnosis, treatment and management of *Haemonchus contortus* in small ruminants. Advances in Parasitology.

[CR3] Charlier J, Van der Voort M, Kenyon F, Skuce P, Vercruysse J (2014). Chasing helminths and their economic impact on farmed ruminants. Trends in Parasitology.

[CR4] Eady SJ, Woolaston RR, Mortimer SI, Lewer RP, Raadsma HW, Swan AA, Ponzoni RW (1996). Resistance to nematode parasites in Merino sheep: sources of genetic variation. Australian Journal of Agricultural Research.

[CR5] González-Garduño R, Torres-Acosta JFJ, Chay-Canul AJ (2014). Susceptibility of hair sheep ewes to nematode parasitism during pregnancy and lactation in a selective anthelmintic treatment scheme under tropical conditions. Research in Veterinary Science.

[CR6] Hansen J, Perry B (1994). The Epidemiology, Diagnosis and Control of Helminth Parasites of Ruminants: A Handbook.

[CR7] Herrera L, Ríos L, Zapata R (2013). Frecuencia de la infección por nemátodos gastrointestinales en ovinos y caprinos de cinco municipios de Antioquia. Revista MVZ Córdoba.

[CR8] Kaplan RM (2020). Biology, Epidemiology, Diagnosis, and Management of Anthelmintic Resistance in Gastrointestinal Nematodes of Livestock. The Veterinary Clinics of North America. Food Animal Practice.

[CR9] Kaplan RM, Vidyashankar AN (2012). An inconvenient truth: global worming and anthelmintic resistance. Veterinary Parasitology.

[CR10] Keyyu JD, Kyvsgaard NC, Monrad J, Kassuku AA (2005). Epidemiology of gastrointestinal nematodes in cattle on traditional, small-scale dairy and large-scale dairy farms in Iringa district, Tanzania. Veterinary Parasitology.

[CR11] Khan MN, Sajid MS, Khan MK, Iqbal Z, Hussain A (2010). Gastrointestinal helminthiasis: prevalence and associated determinants in domestic ruminants of district Toba Tek Singh, Punjab, Pakistan. Parasitology Research.

[CR12] Knox DP (2000). Development of vaccines against gastrointestinal nematodes. Parasitology.

[CR13] McManus C, Do Prado PT, De Melo CB, Brasil BS, Paiva SR (2014). Selection methods for resistance to and tolerance of helminths in livestock. Parasite.

[CR14] Pinilla León JC, Delgado NU, Florez AA (2019). Prevalence of gastrointestinal parasites in cattle and sheep in three municipalities in the Colombian Northeastern Mountain. Veterinary world.

[CR15] Poddar PR, Begum N, Alim MA, Dey AR, Hossain MS, Labony SS (2017). Prevalence of gastrointestinal helminths of sheep in Sherpur, Bangladesh. Journal of Advanced Veterinary and Animal Research.

[CR16] Preston JM, Allonby EW (1979). The influence of breed on the susceptibility of sheep to *Haemonchus contortus* infection in Kenya. Research in Veterinary Science.

[CR17] Raza MA, Iqbal Z, Jabbar A, Yaseen M (2007). Point prevalence of gastrointestinal helminthiasis in ruminants in southern Punjab, Pakistan. Journal of Helminthology.

[CR18] Taylor MA, Coop RL, Wall RL (2016). Veterinary parasitology.

[CR19] Torres-Acosta JFJ, Mendoza-De Gives P, Aguilar-Caballero AJ, Cuellar-Ordaz JA (2012). Anthelmintic resistance in sheep farms: update of the situation in the American continent. Veterinary Parasitology.

[CR20] Van Wyk JA, Bath GF (2002). The FAMACHA© system for managing haemonchosis in sheep and goats by clinically identifying individual animals for treatment. Veterinary Research.

[CR21] Van Wyk JA, Cabaret J, Michael LM (2004). Morphological identification of nematode larvae of small ruminants and cattle simplified. Veterinary Parasitology.

[CR22] Wang T, Ma G, Ang C-S, Korhonen PK, Stroehlein AJ, Young ND, Hofmann A, Chang BCH, Williamson NA, Gasser RB (2020). The developmental phosphoproteome of *Haemonchus contortus*. Journal of Proteomics.

[CR23] Williams EG, Brophy PM, Williams HW, Davies N, Jones RA (2021). Gastrointestinal nematode control practices in ewes: identification of factors associated with application of control methods known to influence anthelmintic resistance development. Veterinary Parasitology: Regional Studies and Reports.

[CR24] Woolaston RR, Baker RL (1996). Prospects of breeding small ruminants for resistance to internal parasites. International Journal for Parasitology.

[CR25] Zajac AM, Garza J (2020). Biology, Epidemiology, and Control of Gastrointestinal Nematodes of Small Ruminants. Veterinary Clinics: Food Animal Practice.

